# Functional Roles of Aromatic Residues and Helices of Papiliocin in its Antimicrobial and Anti-inflammatory Activities

**DOI:** 10.1038/srep12048

**Published:** 2015-07-09

**Authors:** Eunjung Lee, Jin-Kyoung Kim, Dasom Jeon, Ki-Woong Jeong, Areum Shin, Yangmee Kim

**Affiliations:** 1 Department of Bioscience and Biotechnology, Konkuk University, Seoul 143-701, South Korea

## Abstract

A cecropin-like peptide, papiliocin, isolated from the swallowtail butterfly *Papilio xuthus,* possesses high selectivity against gram-negative bacteria. Since Trp^2^ and Phe^5^ are highly conserved residues in cecropin-like peptides, we investigated the role of Trp^2^ and Phe^5^ in antibacterial activity. Substitution of Trp^2^ and Phe^5^ in papiliocin with Ala (papiliocin-2A and papiliocin-5A) revealed that Trp^2^ is a key residue in its antibacterial activities. In order to understand the structural requirements for papiliocin function and to design shorter, but more potent, peptide antibiotics, we designed papiliocin constructs, PapN (residues Arg^1^-Ala^22^ from the N-terminal amphipathic helix). PapN exhibited significant broad-spectrum antibacterial activities without cytotoxicity. Bactericidal kinetics of peptides against *E.coli* showed that papiliocin completely and rapidly killed *E.coli* in less than 10 minutes at 2× MIC concentration, while papiliocin-2A and papiliocin-5A killed four times more slowly than papiliocin. The PapN series peptides permeabilized bacterial membranes less effectively than papiliocin, showing no antibacterial activities in an hour. The results imply that the Trp^2^ and Phe^5^ in the amphipathic N-terminal helix are important in the rapid permeabilization of the gram-negative bacterial membrane. The hydrophobic C-terminal residues permeabilize the hydrophobic bacterial cell membrane synergistically with these aromatic residues, providing selectivity against gram-negative bacteria.

Increasing antibiotic resistance resulting from the widespread use of antibiotics has prompted the need for a novel antimicrobial agent that has not been exposed to microorganisms^1^,^2^. Antimicrobial peptides (AMPs) are antibiotic candidates because of their broad-spectrum antibacterial, antifungal, and antiviral activities. To date, more than 1500 AMPs have been discovered in many species ranging from plants and insects to vertebrates^3^. These molecules are increasingly recognized as significant components of innate immunity in almost all living organisms^4^,^5^,^6^. Although the detailed mechanisms of their action are not well understood, their mode of antibiotic action involves depolarization or permeabilization of the bacterial cell membrane; additionally, some AMPs can traverse intact membranes to interact with intracellular targets^7^,^8^,^9^,^10^.

More than half a million patients suffer from sepsis caused by the presence of pathogens or toxins in the blood every year in North America^11^. Because the systemic inflammatory response is involved in sepsis, treatments involving modulation or inhibition of inflammatory mediators may improve the survival of patients infected with gram-negative and gram-positive bacteria^11^. The release of lipopolysaccharide (LPS) from the main outer membrane part of gram-negative bacteria can cause inflammation and septic shock. Lipoteichoic acid (LTA), a major component of gram-positive bacterial cell walls, may also contribute to sepsis and has polyanionic and lipidic properties similar to LPS in gram-negative bacteria^12^,^13^. Some cationic AMPs have been shown to be effective in killing gram-negative bacteria by binding to polyanionic LPS^14^.

Cecropins belong to a large family of cationic α-helical AMPs identified in a broad spectrum of organisms, including gram-positive bacteria, gram-negative bacteria, and fungi^15^,^16^. Among the insect-derived AMPs, cecropin was the first α-helical form discovered (in 1981) and isolated from the hemolymph of the *Cecropia* moth^17^,^18^. A number of cecropin-like peptides composed of 31–42 amino acids have been identified. As shown in Fig. 1, insect cecropin-like peptides are highly positively charged AMPs and share over 50% sequence similarity, excluding moricin. Most cecropin-like peptides in insects have one or two aromatic residues near the N-terminus. The aromatic residues Trp^2^ and Phe^5^, in particular, are highly conserved. Tertiary structures have revealed that these peptides are generally composed of an N-terminal amphipathic α-helix and a hydrophobic C-terminal α-helix linked by a hinge region^19^,^20^,^21^,^22^,^23^,^24^,^25^. Papiliocin is a novel insect cecropin expressed in the larvae of the swallowtail butterfly, *Papilio xuthus*^26^. Papiliocin shares over 78% sequence identity and over 94% sequence similarity with cecropin A from *Hyalophora* and *Bombyx*. In our previous study, we showed that papiliocin has high bacterial cell selectivity, particularly against gram-negative bacteria, and potent anti-inflammatory activity. We also determined the structure of papiliocin by NMR spectroscopy. The structure revealed that papiliocin contains an N-terminal α-helix (residues Lys^3^–Lys^21^) and a C-terminal hydrophobic α-helix (Ala^25^–Val^35^), linked by a hinge region^27^.

Much work has focused on the role of positively-charged residues in AMPs^28^,^29^,^30^,^31^, but relatively little effort has focused on the role of aromatic residues^31^,^32^. It has been reported that Phe and Trp interact with the acyl chains of the lipid bilayer and are therefore important in membrane permeabilization^31^,^32^. Therefore, it is important to evaluate systematically the role of Trp and Phe residues in antimicrobial activities of AMPs. To understand further the structural requirements for papiliocin function and to design potent and short peptide antibiotics, we designed papiliocin analogs, PapN, composed of residues Arg^1^–Ala^22^ from the amphipathic N-terminal helix and PapC, composed of residues Ala^25^–Lys^37^ from the C-terminal hydrophobic helix. The antimicrobial, anti-inflammatory, and cytolytic activities, as well as mode of action were examined. The ability of peptides to permeabilize and depolarize biological membranes was investigated using intact *E.coli*, *E.coli* spheroplasts, the *E.coli* outer membrane, as well as a model membrane. The anti-inflammatory activities of the peptides were examined by inhibiting nitrite production in LPS-stimulated RAW264.7 cells. Furthermore, to examine the structure–function relationships underlying antimicrobial and anti-inflammatory activities, we investigated the interactions between the peptides and LPS using saturation transfer difference (STD)-NMR.

## Results

### Peptide design

**Figure 1:**
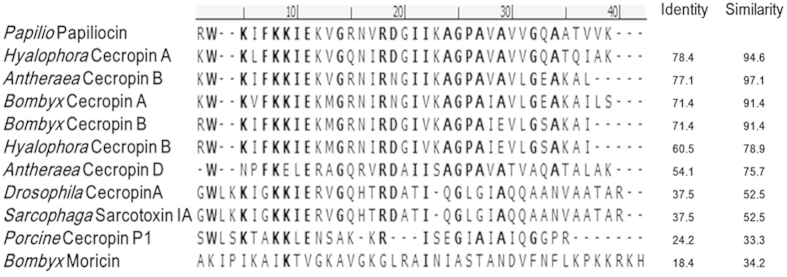
Comparison of 11 known cecropin sequences from ten insects and one mammal. Conserved residues among papiliocin and at least seven other cecropins are in bold. Sequences are arranged according to percent sequence identity to papiliocin to obtain maximal homology (top sequence has highest percent identity).

To elucidate the functional significance of the highly conserved aromatic residues in the N-terminal helix (Fig. 1), Trp^2^ or Phe^5^ was replaced with Ala in a series of papiliocin analogs: papiliocin-2A contains the W2A mutation; papiliocin-5A contains the F5A mutation; and papiliocin-2A5A contains both the W2A and F5A mutations. To develop more potent short peptide antibiotics, we designed an N-terminal analog (PapN), composed of N-terminal helix residues Arg^1^–Ala^22^, and a C-terminal analog (PapC), composed of C-terminal helix residues Ala^25^–Lys^37^. The flexible hinge regions (Gly^23^ and Pro^24^), at the center of papiliocin were excluded from both PapN and PapC. We also substituted aromatic residues with Ala in a series of PapN analogs (PapN-2A, PapN-5A, and PapN-2A5A, Table 1). To examine the effect of Trp^2^ and Phe^5^ positioning on the biological activity of papiliocin, we designed PapN-2F5W, in which the two aromatic residues of PapN were swapped. Deletion of the hydrophobic C-terminal region from papiliocin caused all the PapN analogs to be primarily hydrophilic. On the other hand, PapC is markedly more hydrophobic than papiliocin. PapN is highly cationic with seven positively residues, while PapC has only one positively charged residue.

### CD measurements

**Figure 2:**
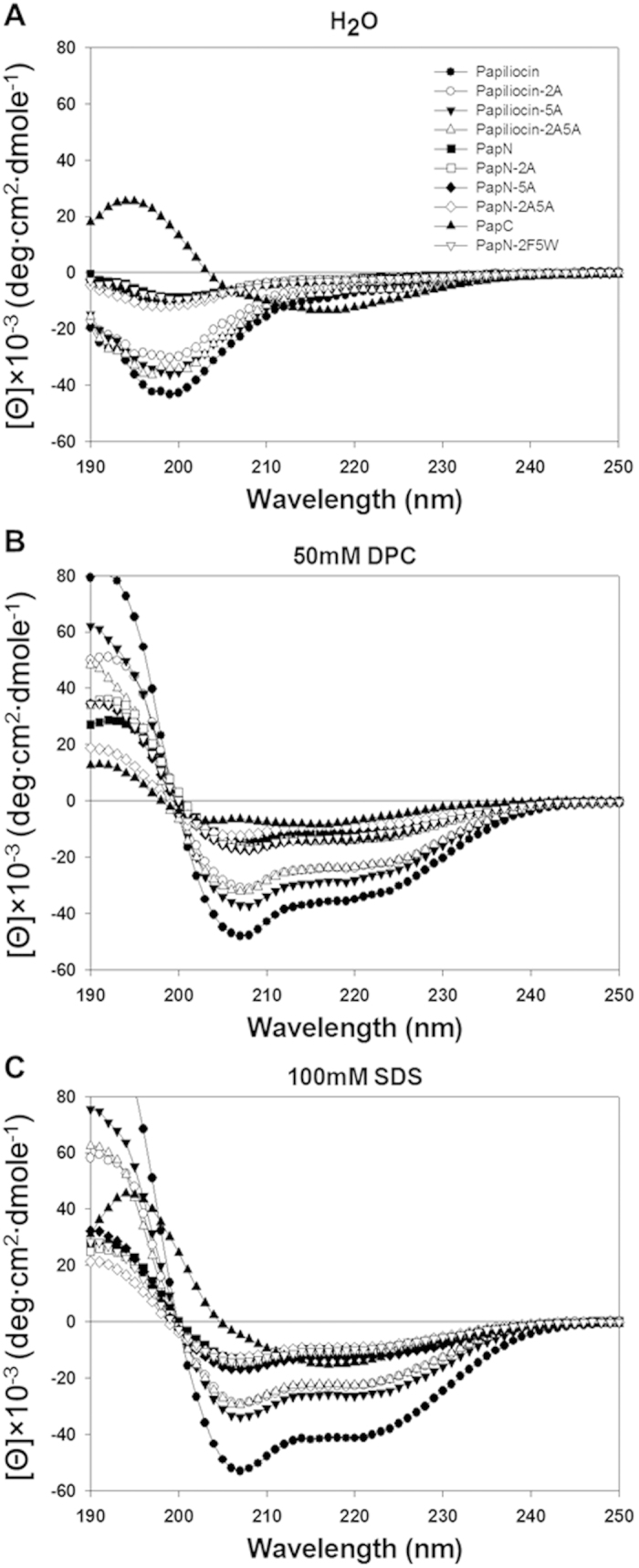
CD spectra of the peptides in (**A**) aqueous solution, (**B**) 50 mM dodecylphosphocholine (DPC) micelles and (**C**) 100 mM SDS micelles. Concentrations of the peptides were 50 μM at pH 4.1.

To examine the secondary structures of the designed papiliocin analogs in membrane-like environments, we analyzed the peptides using CD measurements. The peptides were dissolved in a variety of membrane-mimicking conditions. As shown in Fig. 2, except for PapC, the peptides were unordered in aqueous solution, but they exhibited conformational changes in SDS and DPC micelles. In these membrane-mimetic environments, papiliocin and its N-terminal analogs exhibit characteristic double-negative maxima at 205 nm and 220 nm, implying that they adopt a significant contents of α-helical structure. Papiliocin exhibited much higher α-helical content than papiliocin-2A, papiliocin-5A, or papiliocin-2A5A, implying that the Trp^2^ and Phe^5^ residues at the N-terminal helix confer a more folded structure in membrane-mimetic environments. Papiliocin-5A, which contains Trp^2^, had much higher α-helical content than papiliocin-2A or papiliocin-2A5A, implying that Trp^2^ plays a more prominent role in stabilizing α-helical structure in a lipid environment than Phe^5^. All the PapN series analogs produce similar CD spectra in SDS and DPC micelles, and they have markedly lower α-helical content than papiliocin. PapC produces a broad negative maximum near 215 nm in all environments tested. These results are consistent with PapC adopting a stable β-sheet structure.

### Antimicrobial activity

The antimicrobial activities were examined against a standard set of bacterial strains, including three gram-negative (*E. coli*, *S. typhimurium*, and *P. aeruginosa*) and three gram-positive (*B. subtilis*, *E. faecalis*, and *S. aureus*) species, and were compared with the antimicrobial activities of melittin, which is known to have high antibacterial activity against all of these bacterial strains (Table 2). Minimum inhibitory concentration (MIC) can be defined as the lowest concentration where peptide inhibits the growth completely after a 16 h incubation. In the case of the papiliocin mutants (papiliocin-2A, papiliocin-5A, and papiliocin-2A5A), CD results suggest that substitution of Trp^2^ or Phe^5^ with Ala decreases the interaction of aromatic side chains with phospholipids. However, despite these changes, the papiliocin series of peptides produced an average geometric mean (GM) of MIC values similar to that of papiliocin. PapN retained its antimicrobial activity, but it was 4-fold less potent than papiliocin against gram-negative bacteria. On the other hand, substitution of Trp^2^ or Phe^5^ with Ala drastically attenuated the antibacterial activities of the PapN series of peptides. The antibacterial activities of the peptides against gram-negative bacteria tended to decrease in the following order: Papiliocin = Papiliocin-2A = Papiliocin-5A (0.417) > Papiliocin-2A5A (0.667) > PapN = PapN-5A (1.67) > PapN-2A (3.33) > PapN-2A5A (4.67). These results suggest that the C-terminal helix plays an important role in antibacterial activity against gram-negative bacteria. Substitution of the aromatic residues with alanine only slightly affected MICs when an intact C-terminal helix was present, as in the case of the papiliocin series. However, in the PapN series, which do not have the C-terminal helix, substitution of the aromatic residues with alanine decreased the MICs markedly.

Papiliocin was more effective against gram-negative bacteria than against gram-positive bacteria. PapN was more effective against gram-positive bacteria than papiliocin. For gram-positive bacteria, Papiliocin-2A5A and PapN-2A5A, in which Trp^2^ and Phe^5^ have been substituted, respectively, produced dramatically lower antibacterial activities than the native versions. The antibacterial activities of the analogs against gram-positive bacteria decreased in the following order: PapN (26.7) > papiliocin (37.3) > PapN-5A (53.3) > PapN-2A (64.0) > Papiliocin-2A = Papiliocin-5A (107) > Papiliocin-2A5A = PapN-2A5A (213). This result suggests that the N-terminal aromatic residues play a crucial role in promoting antibacterial activity against gram-positive bacteria, and that the C-terminal helix is not particularly important.

PapN-2A5A was approximately 3-fold less potent against gram-negative bacteria than PapN, and 8-fold less effective against gram-positive bacteria than PapN. PapN-2F5W, containing a Phe at position 2 and a Trp at position 5, produced similar antimicrobial activities against both gram-negative and gram-positive bacteria as PapN, suggesting that exchanging the two aromatic residues does not affect the antibacterial activity of PapN. PapC, which has +2 net charge, produced no antibacterial activity against either gram-negative or gram-positive bacteria, possibly because of a lack of amphiphilic properties and low cationicity, which does not favor interaction with the negatively charged phospholipid membranes of bacteria or other microorganisms. Together, these results suggest that both the N-terminal helix (which contains Trp^2^ and Phe^5^) and the additional C-terminal hydrophobic helix of papiliocin play important roles in maintaining antibacterial potency against both gram-negative and gram-positive bacteria.

We examined the antimicrobial activities of the peptides against multidrug-resistant bacterial strains, including four gram-negative species (*S. typhimurium* 8009*(R)*, *E. coli* 1229(*R)*, *P. aeruginosa* 2095*(R),* and *A. baumannii* 12035*(R)*; Table 3). The papiliocin series peptides produced slightly lower antibacterial activities against multidrug-resistant gram-negative bacteria than papiliocin. Additionally, PapN and PapN-2F5W produced potent antimicrobial activities against multidrug-resistant gram-negative bacteria; these peptides are more potent than melittin. Because PapN is shorter than papiliocin by 15 residues, PapN may have applications as a cost-efficient peptide antibiotic.

### Time-killing kinetics of *E.coli*

**Figure 3:**
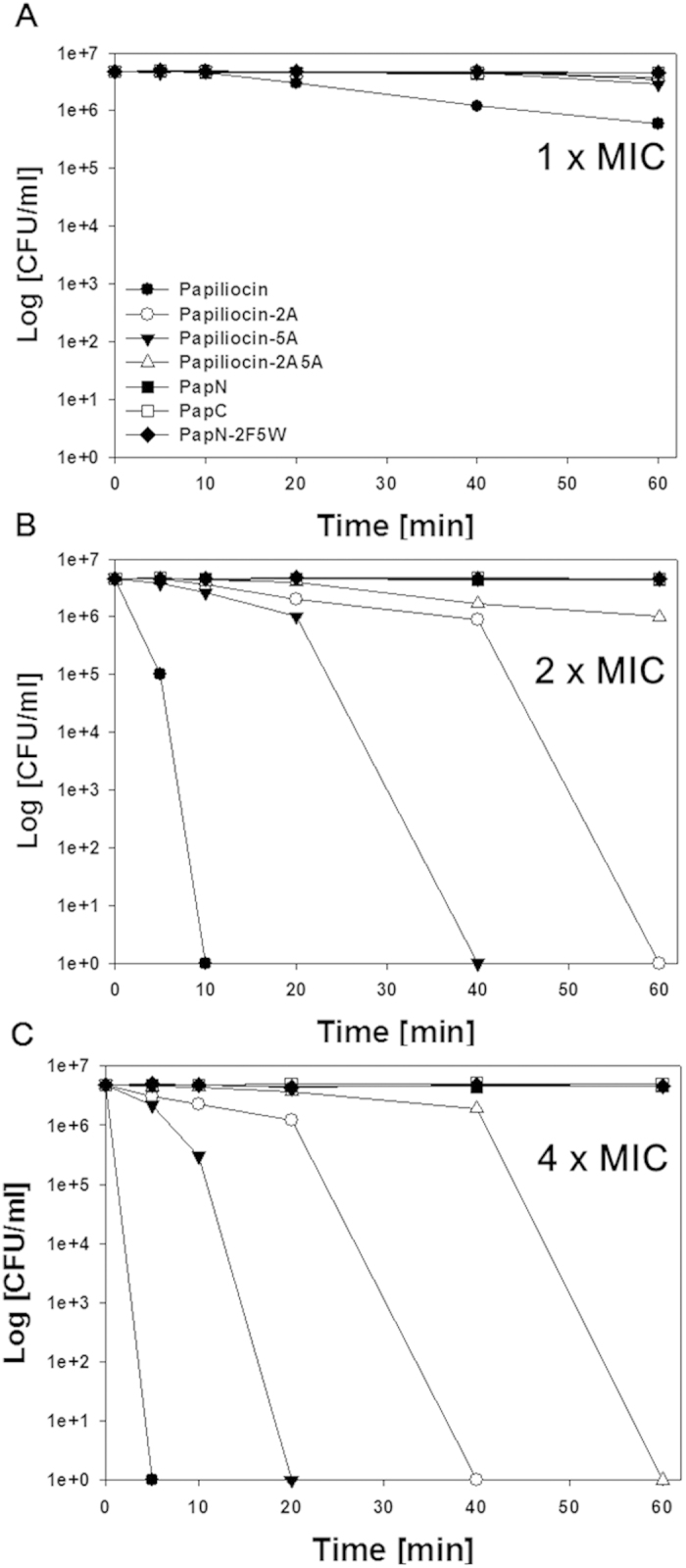
Time-kill kinetics of *Eschericia coli* using papiliocin and its analogs. Concentration-dependent bactericidal curve of *E.coli* using peptides at (**A**) 1× MIC, (**B**) 2× MIC, and (**C**) 4× MIC.

Since papiliocin showed high bacterial selectivity against gram-negative bacteria, bactericidal kinetics of the peptides against gram-negative bacteria was studied by time-killing analyses using *E. coli* (KCTC 1682) at 1× MIC, 2× MIC, and 4× MIC after 5, 10, 20, 40 or 60 min of exposure to the peptides at 37 °C. As shown in Fig. 3, papiliocin completely killed *E. coli* in less than 10 min at 2× MIC. In one hour, papiliocin killed bacteria four times faster than papiliocin-5A at 2× MIC and significantly faster than papiliocin-2A and papliocin-2A5A, implying that Trp^2^ is important in rapid bactericidal activities of papiliocin. The PapN series peptides and PapC did not detectably kill bacteria within an hour, even at 4× MIC. Even though PapN is much slower than papiliocin, it has potent antibacterial activity, with an MIC of 2 uM against *E.coli*. It can be concluded that Trp^2^ and Phe^5^ contribute to rapid permeabilization of the cell membrane within an hour and that the C-terminal helix works synergistically with the N-terminal helix.

### Hemolytic activity and cytotoxicity against mammalian cells

**Figure 4:**
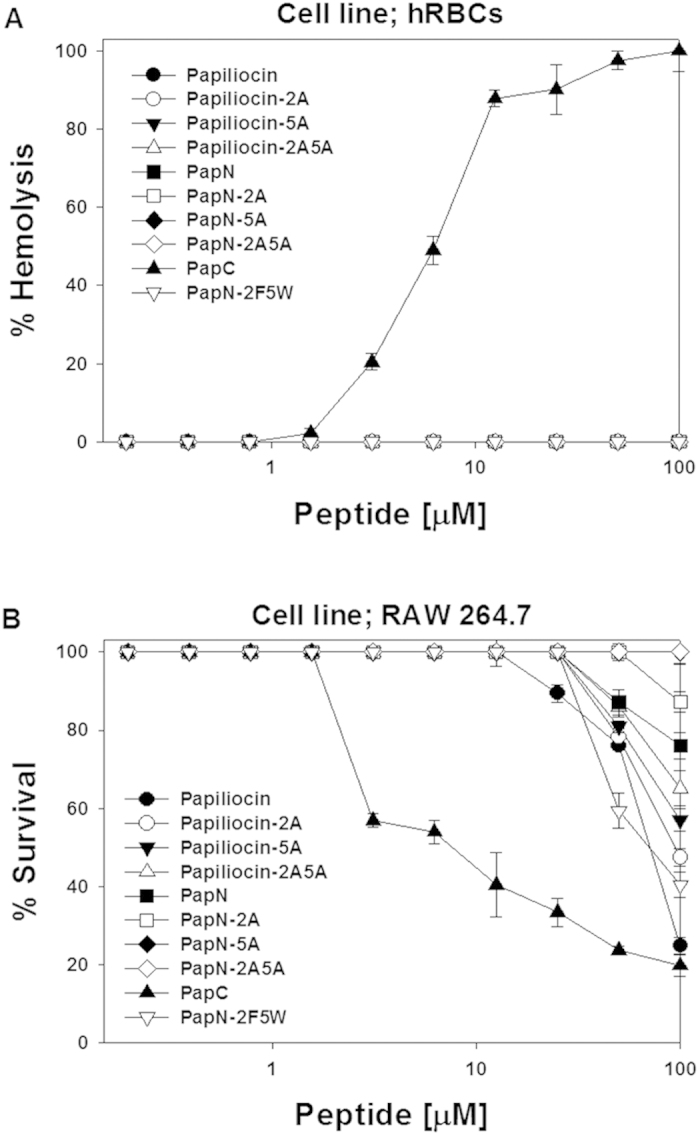
Dose-dependent toxic activity of papiliocin and its analogs toward (A) human RBCs and (B) mouse macrophage-derived RAW264.7 cells. All values represent the means ± standard deviations of three independent experiments.

We examined the cytotoxicity against human erythrocytes. Figure 4A shows the dose-dependent curves of hemolytic activities of the peptides. Papiliocin and the PapN series do not show hemolytic activity, even at 100 μM, while PapC produced 49% hemolytic activity, even at a low concentration of 6.25 μM. Next, we examined the cytotoxicity of the peptides against RAW264.7 macrophage cells. We assessed the effects of the peptides on cell growth by measuring mitochondrial reduction of MTT to a colored product in live cells. Melittin exhibited high cytotoxicity at its MIC, whereas papiliocin and the PapN series were not toxic at their MICs (Fig. 4A,B). The survival rates of RAW264.7 cells in the presence of 50 μM papiliocin and PapN were approximately 76.1% and 86.1%, respectively. All the PapN peptides were less toxic than papiliocin. On the other hand, PapC produced high cytotoxicity against RAW264.7 cells, even at low concentrations. The survival rate of RAW264.7 cells was only 44.2% in the presence of 10 μM PapC. Notably, the C-terminal helix region is a critical determinant of the cytotoxicity of papiliocin. Substituting Ala for Trp^2^ or Phe^5^ in papiliocin resulted in more than a 20% decrease in cytotoxicity against RAW264.7 cells at 100 uM papiliocin. In case of PapN-2A, Pap-5A, and Pap-2A5A, the cell survival rates at a peptide concentration of 100 μM were approximately 90% or more. Thus, Trp^2^ and Phe^5^ are important for the cytotoxic activity of papiliocin toward RAW264.7 cells.

### Dye leakage from model membrane

The mechanism of action of papiliocin and its analogs were examined by measuring their membrane-permeabilizing ability in a model membrane. We monitored calcein release from LUVs of different compositions. Dose-response curves of peptide-induced calcein release revealed that papiliocin-5A is as effective as papiliocin in permeabilizing negatively charged vesicles (7:3 [w/w] EYPC/EYPG) mimicking a bacterial membrane. Compared to papiliocin-5A and papiliocin, papiliocin-2A and papiliocin-2A5A were markedly less effective, implying that Trp^2^ is critical for the membrane permeabilizing activity of papiliocin analogs. The papiliocin series permeabilize into negatively charged vesicles effectively with rapid leakage at very low concentrations, and the percentage of dye leakage is retained beyond 4 μM. At 8 μM, papiliocin produced 100% dye leakage, while papiliocin-5A, papiliocin 2A, and papiliocin-2A5A produced 95.7%, 82.7%, and 53.0% dye leakage, respectively.

**Figure 5:**
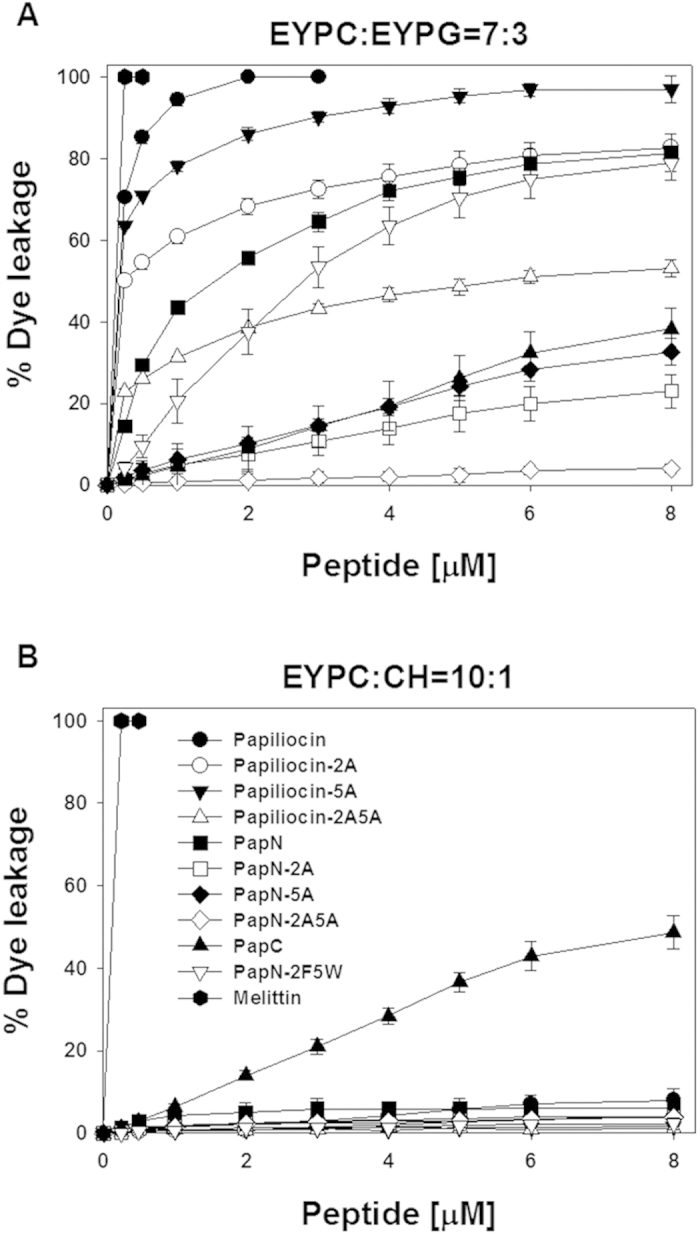
Dose-response curves of calcein leakage from (**A**) egg yolk phosphatidylcholine (EYPC)/egg yolk phosphatidylglycerol (EYPG) (7:3, w:w) and (**B**) EYPC/cholesterol (CH) large unilamellar vesicles (LUVs; 10:1, w-w) induced by the peptides. Calcein leakage was measured 2 min after the addition of papiliocin. The lipid concentration used in the leakage experiments was 64 μM.

PapN and PapN-2F5W were less effective than papiliocin in permeabilizing negatively charged vesicles (7:3 [w/w] EYPC/EYPG) (Fig. 5A). Both peptides caused dye leakage gradually until a concentration of 8 μM was attained (approximately 75%). PapN-5A (PapN Phe^5^ → Ala) was more effective than PapN-2A (Trp^2^ → Ala), suggesting that Trp^2^ is more important than Phe^5^ for permeabilizing negatively charged vesicles, as well as for antimicrobial activity. Furthermore, PapN-2A and PapN-5A were markedly less effective than peptides containing both Trp^2^ and Phe^5^ in permeabilizing negatively charged vesicles. PapN-2A5A produced almost no leakage, implying that both the aromatic residues and the C-terminal hydrophobic helix are important for membrane permeabilization. The membrane-disrupting activity of the peptides against negatively charged vesicles (7:3 [w/w] EYPC/EYPG) decreased in the following order: papiliocin > papiliocin-5A > papiliocin-2A > PapN > PapN-2F5W > papiliocin-2A5A > PapN-5A > PapN-2A > PapN-2A5A (Fig. 5A).

None of the peptides containing the N-terminal helix permeated zwitterionic vesicles (10:1 [w/w] EYPC/CH) effectively. Zwitterionic vesicles mimic the outer leaflet components of human erythrocytes. This result suggests that papiliocin and the PapN series of analogs exhibit selectivity toward bacterial cells. However, Pap-C permeabilized zwitterionic vesicles effectively. Notably, the ability of PapN-2A5A to permeabilize both negatively charged and zwitterionic vesicles was very low, implying that the mechanism of action of PapN-2A5A might be different. The membrane-permeabilizing activities of PapN and Pap-2F5W were similar, suggesting that the position of the Trp residue at either Trp^2^ or Trp^5^ is not important for initial membrane permeabilization. These data are in contrast with those obtained for PapC and melittin, which produced membrane-disrupting activities against both negatively charged and zwitterionic vesicles (Fig. 5A,B).

### Transmembrane potential depolarization assay with *E. coli*

**Figure 6:**
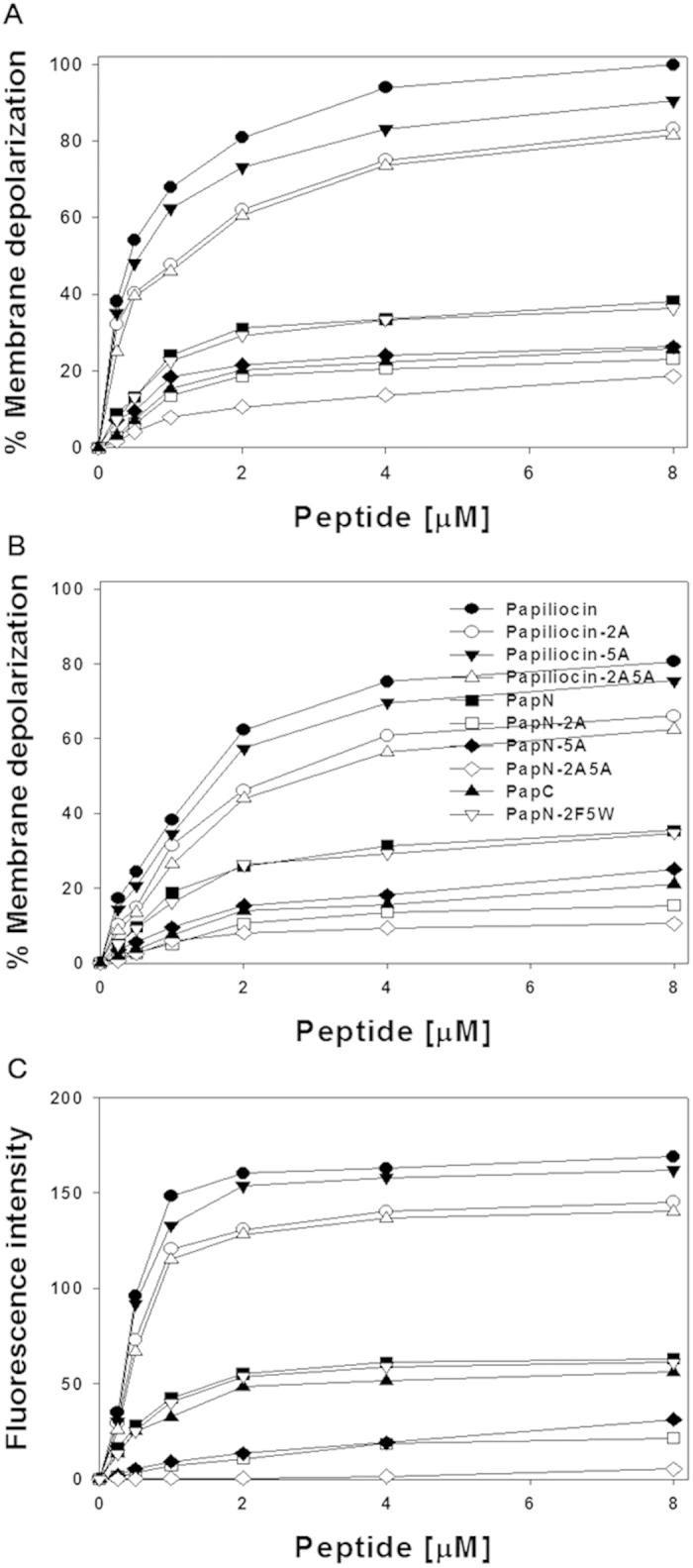
(**A**) Concentration-dependent membrane depolarization of intact *E. coli* KCTC 1682, monitored as an increase in diSC_3_-5 fluorescence in the presence of papiliocin analogs. (**B**) Concentration-dependent membrane depolarization of *E. coli* spheroplasts (ATCC 25922) monitored as an increase in diSC_3_-5 fluorescence in the presence of papiliocin analogs. (**C**) Concentration-dependent permeabilization of the outer membrane of *E. coli* ML35p monitored as an increase in NPN fluorescence intensity in the presence of papiliocin analogs.

Since papiliocin was selective against gram-negative bacteria, we investigated the ability of the peptides to depolarize the gram-negative bacterial membranes using intact *E. coli* (Fig. 6A) and *E. coli* spheroplasts (Fig. 6B). The fluorophore diSC_3_-5 is distributed in the cells and medium, depending on the membrane potential, and self-quenches if concentrated inside bacterial cells. The ability of peptide to depolarize the membrane is indicated by an increase of the fluorescence of diSC_3_-5. As shown in Fig. 6A, papiliocin induced a significant rapid membrane depolarization against intact *E. coli* in a concentration dependent manner. Papiliocin and papiliocin 5A depolarized an intact *E. coli* membrane by 68% and 61%, respectively, at 1 μM (4× MIC). However, papiliocin-2A, as well as papiliocin-2A5A, which lack Trp^2^, shows depolarized intact *E. coli* membrane by only 45% at 1 μM, implying that Trp^2^ is more important in permeabilizing the outer membrane of *E.coli* than Phe^5^. The membrane depolarization ability of the peptides decreased in the following order: papiliocin > papiliocin-5A > papiliocin-2A > papiliocin-2A5A > PapN > PapN-2F5W > PapN-5A > PapN-2A > PapN-2A5A (Fig. 5A). Since PapN and PapN-2F5W behaved similarly, exchanging the two aromatic residues does not affect the depolarization ability of PapN.

Outer membrane of gram-negative bacteria is composed of LPS in the outer leaflet and phospholipids in the inner leaflet. In the outer leaflet, LPS is composed of hydrophilic O-antigen polysaccharide, core oligosaccharide, and lipid A. Lipid A is a lipid component of LPS. Gram-negative bacteria have a peptidoglycan layer between the outer membrane and inner cytoplasmic membrane. *E. coli* (ATCC 25922) spheroplasts were created by removing LPS and peptidoglycan from the *E. coli* outer membrane. Papiliocin and papiliocin-5A produced 10 ~ 20% less depolarization in *E. coli* spheroplasts than in intact cells, while there was no difference in depolarization between spheroplasts and intact cells for the other peptides tested. Therefore, Trp^2^ is important in the interaction with LPS in the outer membrane. PapN series peptides without the C-terminal helix produced less than half depolarization compared to that achieved by papiliocin, in either intact *E. coli* or in *E. coli* spheroplasts.

### Outer membrane permeabilization

Peptide permeanilization of the outer membrane of *E. coli* was evaluated by fluorescence-based 1-N-phenylnapthylamine (NPN) uptake (Fig. 6C). Because NPN exhibits fluorescence weakly in an aqueous environment, but strongly in the hydrophobic interior of a lipid bilayer, destabilization of the outer membrane by peptides cause the dye to enter the damaged membrane, resulting in increasing fluorescence. The fluorescence from *E. coli* (KCTC 1682) was monitored after incubation with NPN and the peptides. Similar to the results obtained from membrane depolarization assay, papiliocin and papiliocin-5A induced greater NPN uptake than papiliocin-2A or papiliocin 2A5A, which do not have Trp^2^. This result implies that Trp^2^ is more important in membrane permeabilization than Phe^5^, probably through interactions with hydrophobic fatty acid chains of lipid A. The PapN series peptides were less active than papiliocin series peptides. Outer membrane permeabilization of the peptides from the NPN uptake experiment decreased in the following order: papiliocin > papiliocin-5A > papiliocin-2A > papiliocin-2A5A > PapN > PapN-2F5W > PapN-5A > PapN-2A > PapN-2A5A.

All PapN analogs produced less than half the extent of membrane depolarization of intact *E. coli*, *E. coli* spheroplasts, and outer membrane permeabilization than papiliocin, implying that the C-terminal helix may be important for its hydrophobic interaction with lipid A in the outer membrane of *E.coli*, as well as hydrophobic phospholipids in the inner membrane. Even though PapN-2A5A has antibacterial activity against *E.coli*, PapN-2A5A did not cause permeability of the inner or outer membrane, implying that it kills bacteria via a different bactericidal mechanism.

### Inhibition of NO production in LPS-stimulated RAW264.7 cells

**Figure 7:**
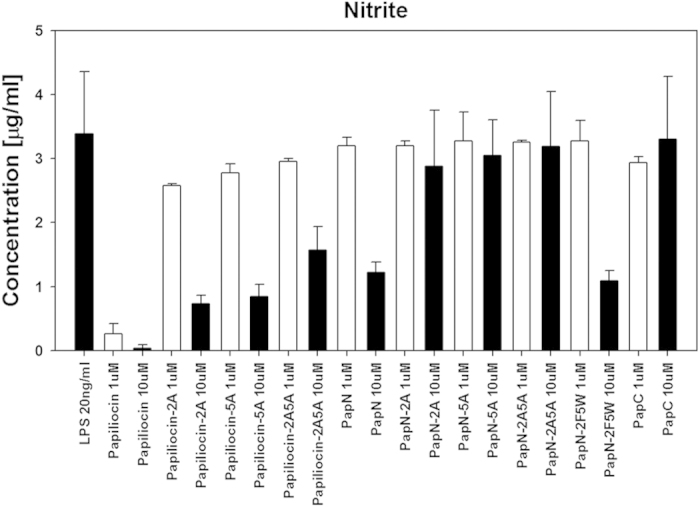
Anti-inflammatory activities of papiliocin and its analogs in LPS-stimulated RAW264.7 cells. Inhibition of nitrite production by papiliocin and its analogs.

When released from gram-negative bacteria, LPS causes septic shock by inducing the production of pro-inflammatory cytokines and NO^33^. To examine the structural mechanism of the anti-inflammatory activity of papiliocin, we indirectly measured inhibition of NO production by the peptides in LPS-simulated RAW264.7 macrophages. As shown in Fig. 7, all the papiliocin series of peptides, which contain both the N- and C-terminal helices, strongly inhibited NO production in LPS-stimulated RAW264.7 cells. Papiliocin was markedly more efficient than papiliocin-2A, papiliocin-5A, or papiliocin-2A5A, implying that Trp^2^ and Phe^5^ are important for anti-inflammatory activity. Similarly, among the PapN series, only PapN and PapN-2F5W significantly inhibited NO production in LPS-stimulated RAW264.7 cells at a concentration of 10 μM, indicating that the N-terminal helix region, which harbors both aromatic residues, is involved in the inhibition of NO productions. PapC, which contains only the C-terminal helix region, does not inhibit NO production. The activities of PapN and PapN-2F5W were lower than that of papiliocin, implying that the C-terminal helix region of papiliocin may function synergistically with the N-terminal helix. Additionally, PapN-2A, PapN-5A, and PapN-2A5A did not inhibit NO production, implying that both Trp^2^ and Phe^5^ are crucial for inhibiting NO production in the absence of C-terminal helix.

### NMR studies of peptides bound to LPS

**Figure 8:**
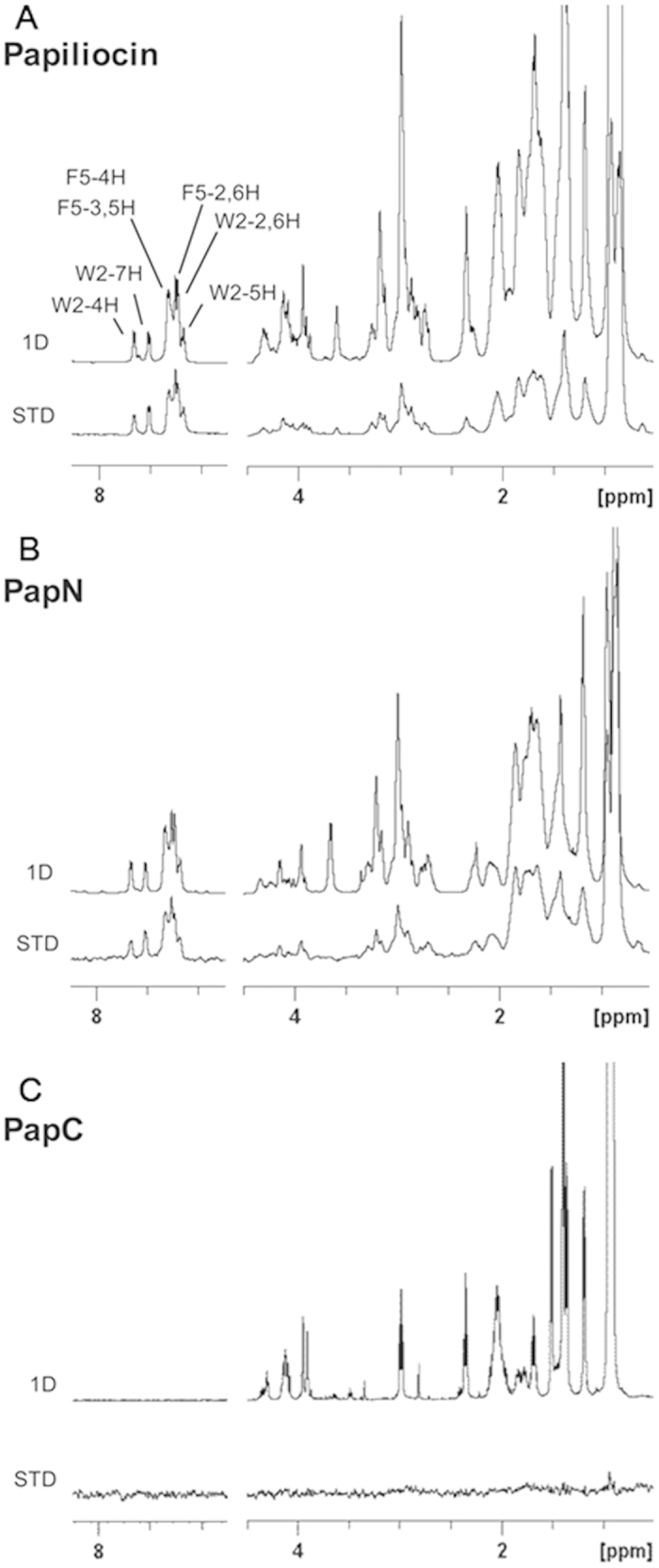
Saturation transfer difference (STD)-NMR results showing the interaction between papiliocin, PapN, and PapC with LPS. (**A**) Papiliocin, (**B**) PapN, and (**C**) PapC. Spectral differences primarily constituted resonances fromn the peptide protons bound to LPS. The STD experiments were carried out using a peptide concentration of 0.5 mM in presence of 0.1 mg/mL LPS in D_2_O, pH 5.9 at 298 K.

To investigate the mode of peptide interaction with LPS, we performed STD-NMR experiments^27^. Figure 8 shows the ^1^H spectra of free peptides and the STD spectra of peptides bound to LPS. The STD spectra of papiliocin and PapN revealed that the aromatic ring protons of Trp and Phe, as well as a number of aliphatic side-chain proton resonances, were in close association with LPS. The aromatic ring protons of Trp and Phe produced a strong STD effect, implying that they are intimately associated with LPS. Surprisingly, the STD effect of PapC by itself was not detected for any region. These data demonstrate the importance of the N-terminal helix in interactions with the LPS of gram-negative bacterial cell membranes. Moreover, PapC by itself does not interact with LPS, resulting in no antibacterial or anti-inflammatory activity.

### FITC-labeled LPS aggregates

**Figure 9:**
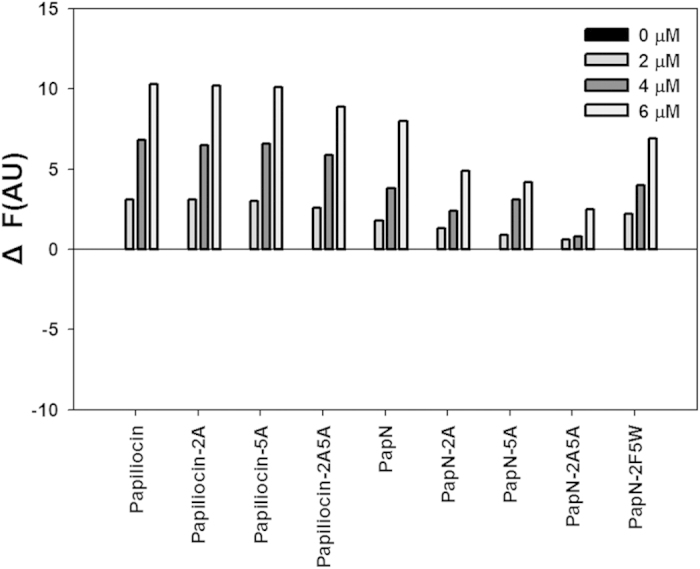
Disaggregation of LPS by the peptides. Enhancement of the intensity of FITC-labeled LPS is shown as a function of the concentration of papiliocin and its analogs.

We examined the ability of papiliocin and PapN analogs to dissociate large LPS aggregates by monitoring the fluorescence intensity of FITC-conjugated LPS^34^,^35^. A dose-dependent increase in FITC-LPS fluorescence was caused by addition of peptides, indicating that interaction with papiliocin analogs and PapN analogs resulted in LPS disassociation (Fig. 9). Generally, papiliocin and the papiliocin series of peptides produced a marked increase in FITC-LPS fluorescence. Papiliocin-2A5A produced a slightly smaller change in FITC-LPS fluorescence intensity than papiliocin, implying that electrostatic interactions between positively charged residues in the amphipathic helix and negatively charged LPS is more important than Trp^2^ and Phe^5^ in LPS dissociation. Although the LPS dissociation activity of PapN was lower than that of papiliocin, PapN and PapN-FW caused a FITC-LPS fluorescence increase of 80% and 65% of papiliocin, respectively. However, PapN-2A and PapN-5A produced only a small increase in FITC-LPS fluorescence, and PapN-2A5A produced very little FITC-LPS fluorescence, implying that Trp^2^ and Phe^5^ may act synergistically with the C-terminal helix in the disaggregation of LPS, thereby mediating anti-inflammatory activities and antibacterial activities against gram-negative bacteria. This result is consistent with the results obtained for the inhibition of NO production and antibacterial activities.

## Discussion

In this study, we have characterized the factors critical for the biological activities of papiliocin. The therapeutic potential of peptide antibiotics lies in their ability to kill bacterial cells effectively without exhibiting cytotoxicity significantly toward mammalian cells. This property is assessed by the therapeutic index which can be defined as a ratio of the minimally effective concentration against human hRBCs to the MIC against bacterial cells. High MHC (i.e., low hemolysis) and low MIC (i.e., high antimicrobial activity) yield a high therapeutic index, indicating that the compound may be a good candidate as an antibiotic. The therapeutic index of the papiliocin mutants (papiliocin-2A, papiliocin-5A, and papiliocin-2A5A) are very similar to each other, and all the papiliocin peptides are highly selective for gram-negative bacteria. The therapeutic index of the N-terminal analogs PapN and PapN-2F5W (both contain two aromatic residues, but no C-terminal helix) were 4-fold less than that of papiliocin (Table 2). However, PapN-2A, PapN-5A, and Pap-2A5A, in which the aromatic residues are substituted with Ala, exhibit much lower antibacterial activities than PapN. Therefore, in the presence of the C-terminal helix, the aromatic residues do not play a significant role in antibacterial activity against gram-negative bacteria. However, in the case of the PapN series, which lack the C-terminal helix, the aromatic residues are crucial for antibacterial activity.

PapN and PapN-2F5W have a higher therapeutic index (33.3 and 21.3) than papiliocin (12.0) against gram-positive bacteria. PapC, which does not contain the N-terminal helix region, shows no antimicrobial activity even at 128 μM against either gram-negative or gram-positive bacteria, except *E. coli*, but shows high cytotoxicity against hRBCs. Thus, the C-terminal helical region of papiliocin is likely related to its ability to kill gram-negative bacteria, though the C-terminal helix is not active on its own. The therapeutic index of the Ala-substituted PapN analogs decreased in the following order against both gram-positive and gram-negative bacteria: PapN ≈ PapN-2F5W > PapN-5A > PapN-2A > PapN-2A5A. PapN-2A and PapN-5A retained their antimicrobial activities although their therapeutic indices were 2- to 4-fold lower than that of PapN against both gram-negative and gram-positive bacteria. However, the therapeutic index of PapN-2A5A was 30-fold lower than that of PapN against gram-positive bacteria and 4-fold lower than that of PapN against gram-negative bacteria. These results imply that both Trp^2^ and Phe^5^ play crucial roles in antimicrobial activity in the absence of the C-terminal helix.

MIC is the lowest concentration of peptide that inhibited growth completely after 16 hour incubation was similar for papiliocin, papiliocin-2A and papiliocin 5A. However, time-killing analysis using *E. coli* showed that papiliocin completely killed *E. coli* much faster than papiliocin 2A or papiliocin 5A. PapN series peptides did not kill within an hour at 4× MIC. Therefore, it can be concluded that Trp^2^ and Phe^5^ contribute to rapid permeabilization into the cell membrane within an hour and the C-terminal helix helps further permeabilization in a synergistic manner.

The interactions between AMPs and LPS and the orientation of the AMPs in lipid bilayers have been examined using a variety of biophysical techniques, including isothermal titration calorimetry, infrared spectroscopy, dynamic light scattering, transferred NOE, STD-NMR, solid-state NMR, and MD simulation^36^,^37^,^38^,^39^,^40^. In this study, STD-NMR experiments have revealed that Trp^2^ and Phe^5^, as well as a number of aliphatic side-chain proton resonances of papiliocin and PapN, produce a high STD effect in the presence of LPS, while PapC itself does not exhibit an STD effect and does not interact with LPS. These data implied that Trp^2^ and Phe^5^ in the amphipathic N-terminal helix play an important role in the interaction with the LPS of gram-negative bacterial cell membranes.

Cytoplasmic membrane-permeabilizing ability was measured using a fluorescent dye entrapped within LUVs or in an *E.coli* membrane. Among all the peptides examined, the papiliocin series induced rapid leakage of dye from negatively charged vesicles which mimic bacterial cell membrane; further, Trp^2^ was more important than Phe^5^ in causing leakage. Interestingly, the membrane-permeabilizing activity of PapN-2F5W is a little lower than that of PapN for negatively charged vesicles and the *E.coli* membrane, suggesting that Trp^2^, in the flexible N-terminal region, may be more effective than Trp^5^, in the helical core region, for cytoplasmic membrane permeabilization. In AMPs, Trp residue located in the peptide hydrophobic core exhibited restricted motion within membranes, and participated mainly in hydrophobic interactions with the acyl chains of phospholipids^31^,^32^,^41^. Furthermore, Trp can interact with the membrane through hydrophilic interaction of the indole ring of the Trp side chain with the carbonyl and phosphate oxygen of LPS to form a hydrogen bond^39^. Also, Trp can interact with the acyl chain of lipid A in LPS. The transmembrane potential depolarization assay, as well as the outer membrane permeabilization assay with *E. coli*, imply that the positively charged residues in the amphipathic N-terminal helix in all peptides interact electrostatically with the polysaccharide and phosphate groups of LPS, while Trp^2^ and Phe^5^ interact primarily with lipid A in LPS. Trp^2^ at the N-terminus plays a critical role in permeabilizing both the inner phospholipid membrane and the outer membrane. The C-terminal hydrophobic helix may enhance membrane permeability in a synergistic manner via hydrophobic interaction with the acyl chain of LPS, a constituent of lipid A in the outer membrane, or the phospholipid in the inner membrane.

Collectively, our data suggest that Trp^2^ and Phe^5^ at the N-terminal amphipathic helix are responsible for the antimicrobial and anti-inflammatory activities of papiliocin. The N-terminal helix region, which harbors the essential Trp and Phe residues, plays a key role in LPS interactions and bacterial cytoplasmic membrane permeabilization. The decreased antimicrobial activities against gram-negative bacteria and reduced anti-inflammatory activities of PapN series peptides may be attributed to decreased interaction between the peptides and LPS in the absence of the hydrophobic C-terminal helix. Recently, a study examining the N-terminal alkylation of lactoferricin-derived peptide LF11 (lauryl-LF11) revealed that lauryl-LF11 had higher antimicrobial activity against the gram-negative bacterium *Salmonella enterica* than LF11, and inhibited strongly LPS-induced TNF-α, resulting in increased endotoxin-neutralizing activities^42^. This study demonstrated that the lauryl-modified peptide possesses a strong hydrophobic component that enhances interaction between the peptide and LPS, although electrostatic interaction also plays a crucial role. Similarly, the amphipathic N-terminal helix of papiliocin, with six Lys and one Arg, plays a crucial role in forming electrostatic interactions with the polysaccharide in LPS, as well as the negatively charged bacterial cell membrane, while the C-terminal helix may contribute to strong hydrophobic interactions with lipid A of LPS.

**Figure 10:**
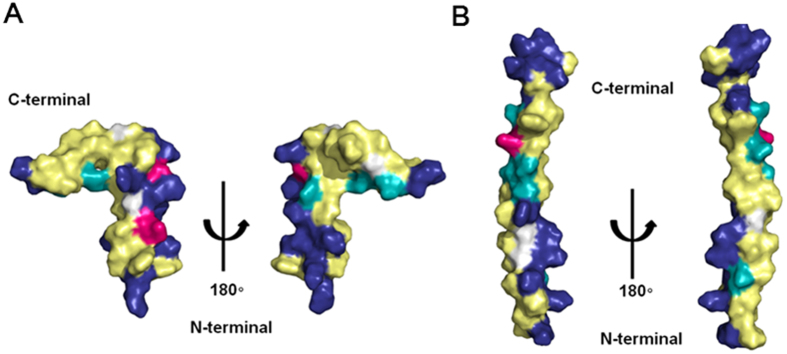
Comparison of the hydrophobic and electrostatic potentials of papiliocin and moricin. (**A**) Hydrophobic and electrostatic potentials of papiliocin (pdb: 2LA2) and (**B**) moricin (pdb: 1 × 22), as determined by NMR spectroscopy. The residues are color-coded as follows: yellow, hydrophobic; cyan, hydrophilic; blue, positive; red, negative; white, neutral.

Papiliocin exhibited low antimicrobial activity against gram-positive bacteria. This is a characteristic feature of AMPs belonging to the cecropin family. In contrast, moricin, a 42-residue insect AMP isolated from the silkworm *Bombyx mori,* had higher antimicrobial activity against gram-positive bacteria than cecropin B, a major AMP of *B. mori*^43^. As shown in Fig. 1, cecropin B shares 71.4% sequence identity with papiliocin. Moreover, similar to papiliocin (Fig. 10A), cecropin B has an amphipathic N-terminal helix and a hydrophobic C-terminal helix^27^,^44^. The solution structure of moricin revealed a straight α-helical structure from residues 5–36 (Fig. 10B)^45^. Interestingly, the N-terminal region (from residues 5–22) of the helix in moricin is amphipathic, while the C-terminal region of the helix (from residues 23–36) is hydrophobic. However, the flexible C-terminal region (from residues 38–41) in moricin, which is not part of the helix, harbors a cluster of four basic amino acid residues (Fig. 10B)^45^. In contrast to other cecropins, porcine cecropin P1 does not exhibit a bent structure; rather, it is composed of one straight helix^46^. However, similar to other cecropins, P1 contains an amphipathic N-terminus α-helix and a C-terminal hydrophobic α-helix; moreover, P1 exhibits lower antimicrobial activity against gram-positive bacteria^46^. Remarkably, the positively charged cluster at the C-terminal region in moricin results in higher antimicrobial activities against gram-positive bacteria that cecropin P1 and papiliocin, suggesting that these four positively charged residues in moricin interact with the surface of gram-positive bacteria with electrostatic interaction and this is important for killing gram-positive bacteria.

Gram-positive and gram-negative bacteria differ essentially in the morphology of their membrane surfaces. In addition to the cytoplasmic membrane, gram-negative bacteria have an outer membrane that is rich in LPS. These bacteria have a peptidoglycan layer between the two membranes, too. On the contrary, peptidoglycan makes up a major part of the cell wall of gram-positive bacteria. Peptidoglycan contains teichoic acid and LTA. The peptidoglycan layer of gram-positive bacteria is approximately ten times thicker (20–80 nm) than that of gram-negative bacteria^47^. The phospholipid composition of cell membranes can differ among bacteria dramatically. In the case of *E. coli* (gram-negative), the major lipid in both leaflets is phosphatidylethanolamine, whereas in gram-positive *B. subtilis*, the major lipids are the anionic phosphatidylglycerol and cardiolipin^48^. Even though PapN (without the C-terminal helix) kills bacteria more slowly than papiliocin, it exhibits significant antibacterial activities against gram-negative and gram-positive bacteria, has anti-inflammatory activity and low cytotoxicity, suggesting that PapN could be a potent peptide antibiotic. Therefore, the lack of electrostatic interaction with the hydrophobic C-terminal helix region in papiliocin may weaken the interactions between papiliocin and gram-positive bacterial cell membranes, but enhance antibacterial activity against gram-negative bacteria.

In conclusion, Trp^2^ and Phe^5^ in the N-terminal amphipathic helical region are essential for the antimicrobial and anti-inflammatory activities of papiliocin. The N-terminal helix is involved in anchoring Trp^2^ and Phe^5^ onto lipid A on LPS and in forming electrostatic interactions between the positively charged residues and the LPS polysaccharide. Trp^2^ is particularly important in rapid permeabilization of the gram-negative bacterial cell membrane, and the hydrophobic portion of the C-terminal helical region may provide bacterial selectivity against gram-negative bacteria membranes. These interactions contribute to its antibacterial and anti-inflammatory activities in a synergistic manner.

## Methods

MICs of the peptides against six standard bacteria and four multidrug-resistant bacteria were determined by a broth microdilution assay as described previously^49^. MIC can be defined as the lowest concentration of peptide that completely inhibited growth after a 16 h incubation. The time-killing kinetics of *E. coli* (KCTC 1682) at 1× MIC, 2× MIC, and 4× MIC after 5, 10, 20, 40 or 60 min of exposure to the peptides was performed at 37 °C to examine the bactericidal kinetics as reported previously^50^. Cytotoxicities against mammalian cells were measured against human RBCs, mouse macrophage-derived RAW264.7 cells, and fibroblast NIH-3T3 cells. The anti-inflammatory activities of the peptides were examined by inhibiting nitrite production in LPS-stimulated RAW264.7 cells^27^.

In order to investigate the mode of action, membrane permeabilization ability was monitored using a model membrane and *E.coli*. Calcein leakage induced by the peptides was measured in egg yolk phosphatidylcholine (EYPC)/egg yolk phosphatidylglycerol (EYPG) (7:3, w/w) and EYPC/cholesterol (CH) large unilamellar vesicles (LUVs; 10:1, w:w) as described previously^27^. The depolarization assay was conducted with intact *E. coli* bacteria (KCTC 1682) as well as *E. coli* spheroplasts (ATCC 25922)^51^,^52^. The ability of peptide to depolarize the membrane is indicated by an increase in the fluorescence of diSC_3_-5. *E. coli* spheroplasts were prepared by removing LPS and peptidoglycan from the *E. coli* (ATCC 25922) outer membrane as reported previously^51^,^52^. The membrane potential was dissipated fully by adding gramicidin D with a final concentration of 0.2 nM. The membrane potential dissipating activity of the peptides can be calculated as follows:


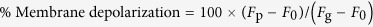


where *F*_p_ is the fluorescence value 5 min after addition of the peptides and *F*_0_ is the fluorescence value after addition of the diSC_3_-5 dye, and *F*_g_ the fluorescence value after the addition of gramicidin D. The outer membrane-permeabilizing activity of peptides into the outer membrane of *E. coli* (KCTC 1682) was determined using the NPN uptake assay^50^.

## Additional Information

**How to cite this article**: Lee, E. *et al.* Functional Roles of Aromatic Residues and Helices of Papiliocin in its Antimicrobial and Anti-inflammatory Activities. *Sci. Rep.* **5**, 12048; doi: 10.1038/srep12048 (2015).

## Figures and Tables

**Table 1:** Amino acid sequences and properties of the peptides.

Peptide	Sequence	MW	Net charge	Hydrophilicity
Papiliocin	RWKIFKKIEKVGRNVRDGIIKAGPAVAVVGQAATVVK-NH_2_	4002.8	8	0.19
Papiliocin-2A	R**A**KIFKKIEKVGRNVRDGIIKAGPAVAVVGQAATVVK-NH_2_	3887.7	8	0.27
Papiliocin-5A	RWKI**A**KKIEKVGRNVRDGIIKAGPAVAVVGQAATVVK-NH_2_	3926.7	8	0.24
Papiliocin-2A5A	R**A**KI**A**KKIEKVGRNVRDGIIKAGPAVAVVGQAATVVK-NH_2_	3811.6	8	0.32
PapN	RWKIFKKIEKVGRNVRDGIIKA-NH_2_	2654.2	7	0.62
PapN-2A	R**A**KIFKKIEKVGRNVRDGIIKA-NH_2_	2539.1	7	0.77
PapN-5A	RWKI**A**KKIEKVGRNVRDGIIKA-NH_2_	2578.1	7	0.71
PapN-2A5A	R**A**KI**A**KKIEKVGRNVRDGIIKA-NH_2_	2463.0	7	0.89
PapN-2F5W	R**F**KI**W**KKIEKVGRNVRDGIIKA-NH_2_	2655.2	7	0.62
PapC	AVAVVGQAATVVK-NH_2_	1268.5	2	−0.55

*Hydrophilicity is the total hydrophilicity (sum of all residue hydrophilicity indices) divided by the number of residues, according to the Hopp and Woods index (66).

**Table 2:** Antimicrobial activities of the peptides against standard bacterial strains.

	MICs^a^	Papiliocin	Papiliocin-2A	Papiliocin-5A	Papiliocin-2A5A	PapN	PapN-2A	PapN-5A	PapN-2A5A	PapN-2F5W	PapC	Melittin
Gram│negative	*Escherichia coli*	0.250	0.250	0.250	0.500	1.00	2.00	1.00	2.00	1.00	128	4.00
	*Pseudomonas aeruginosa*	0.500	0.500	0.500	0.500	2.00	4.00	2.00	8.00	2.00	>128	4.00
	*Salmonella typhimurium*	0.500	0.500	0.500	1.00	2.00	4.00	2.00	4.00	2.00	>128	4.00
	GM^b^	0.417	0.417	0.417	0.667	1.67	3.33	1.67	4.67	1.67	213	4.00
	MHC^c^	200	200	200	200	200	200	200	200	200	1.56	0.391
	Therapeutic Index^d^ (MHC/GM)	480	480	480	480	120	60.0	120	42.9	120	0.007	0.098
Gram│Positive	MICs^a^	Papiliocin	Papiliocin-2A	Papiliocin-5A	Papiliocin-2A5A	PapN	PapN-2A	PapN-5A	PapN-2A5A	PapN-2F5W	PapC	Melittin
	*Bacillus subtilis*	16.0	64.0	64.0	128	8.00	32.0	16.0	>128	8.00	>128	4.00
	*Eenterococcus faecalis*	64.0	128	128	>128	64.0	128	128	>128	64.0	>128	2.00
	*Staphylococcus aureus*	32.0	128	128	>128	8.00	32.0	16.0	128	16.0	>128	4.00
	GM^b^	37.3	107	107	213	26.7	64.0	53.3	213	29.3	256	3.33
	MHC^c^	200	200	200	200	200	200	200	200	200	1.56	0.390
	Therapeutic Index^d^ (MHC/GM)	5.36	1.87	1.87	0.939	7.50	3.13	3.75	0.939	6.82	0.006	0.117

^a^Minimum inhibitory concentrations (MICs) were determined in three independent experiments performed in triplicate with a standard deviation of 14.0%.

^b^The geometric means (GM) of the MIC values from all six bacterial strains are shown. When no antimicrobial activity was observed at 128 μM, a value of 256 μM was used to calculate the therapeutic index.

^c^The minimal peptide concentration that produces hemolysis. When no detectable hemolysis was observed at 100 μM, a value of 200 μM was used to calculate the therapeutic index.

^d^The ratio of the minimum hemolytic concentration (MHC; μM) over the GM of the MIC (μM). Larger values indicate greater cell selectivity.

**Table 3:** Antimicrobial activities of the peptides against multidrug-resistant bacterial strains.

MICs	Papiliocin	Papiliocin-2A	Papiliocin-5A	Papiliocin-2A5A	PapN	PapN-2A	PapN-5A	PapN-2A5A	PapN-2F5W	PapC	Melittin
MDRST 8009(R)	0.500	1.00	1.00	4.00	2.00	2.00	4.00	8.00	2.00	>128	8.00
MDREC 1229(R)	0.500	1.00	1.00	2.00	4.00	2.00	2.00	4.00	2.00	>128	8.00
MDRPA 2095(R)	1.00	1.00	1.00	1.00	2.00	4.00	8.00	16.0	2.00	>128	2.00
MDRAB 12035(R)	0.500	0.500	0.500	0.500	2.00	8.00	8.00	32.0	4.00	>128	4.00

MDRST, multidrug-resistant *Salmonella typhimurium*; MDREC, multidrug-resistant *Escherichia coli*; MDRPA, multidrug-resistant *Pseudomonas aeruginosa*; MDRAB, multidrug-resistant *Acinetobacter baumannii.*
